# Supplementary material for: Physical confinement and phagocytic uptake induce directional migration
Source: bioRxiv. 2025 May 14:2025.05.13.653701. Preprint. [Version 1] doi: 10.1101/2025.05.13.653701 (PMC12478418; doi:10.1101/2025.05.13.653701)
Supplement: 1 [file NIHPP2025.05.13.653701V1-supplement-1.pdf]

bioRxiv preprint doi: https://doi.org/10.1101/2025.05.13.653701; this version posted May 14, 2025. The copyright holder for this preprint (which was not certified by peer review) is the author/funder. This article is a US Government work. It is not subject to copyright under 17 USC 105 and is also made available for use under a CC0 license.

Supplementary Figure 1: Strategy for controlling for bead density. A) Schematic depicting phagocytic bead labelling and the pHrodo tag fluorescing inside cells but not externally. B) Duplication of Figure 2A. Examples of either internalized (blue and purple circles) or external (yellow and green circles) pHrodo-red staining outlined in both composite and pHrodo only images. Scale bar represents 100µm. C) Example phase contrast images displaying different bead densities. No beads (top) through high bead density (bottom). Scale bar represents 50µm. D) Histograms detailing the breakdown of average bead densities per field of view across all experimental runs. Low bead density was classified as any field of view with a bead average between 50 and 500 beads; medium bead density was 500 to 950 beads; high bead density was any field of view average above 950 beads. Low bead densities in confined images most closely matched the density of typical media images.

Supplementary Figure 2: Controlling for agarose stiffness and DMSO influence on motility and phagocytosis. A) Persistence of the cells moving randomly (e.g. not post-phagocytic) in media, under 1% agarose, 2.5% agarose, or 5% agarose. B) Velocity (in µm/min) of cells moving in media, under 1% agarose, 2.5% agarose, or 5% agarose. C) Maximum total accumulated distance traveled (µm) for cells moving in media, under 1% agarose, 2.5% agarose, or 5% agarose. D) Schematic depicting how drugs were added into the agarose. Aliquots of 1% agarose were created and then vehicle or drug was added at the corresponding molarity before the agarose was poured into the confinement wells. E-K) These cells were either treated with water or DMSO. E) The percentage of fluorescent cells in a field of view, normalized to T=0. F) Average phagosome size (µm^2). G) Average phagosome area per phagocytic cell (µm^2). H) Average number of phagosomes per phagocytic cell. I) The persistence of the cell during the length of its track. J) Velocity of the cells migrating (µm/min). K) The maximum accumulated distance (µm) that the cell traveled during its tracking. L-O) These cells were either treated with DMSO or CK-666. These graphs relate to the experiments presented in Figure 3. L) Average phagosome area per phagocytic cell (µm^2). M) Average number of phagosomes per phagocytic cell. N) Velocity of the cells migrating (µm/min). O) The maximum accumulated distance (µm) that the cell traveled during its tracking. For all graphs, black points demonstrate experiment

bioRxiv preprint doi: https://doi.org/10.1101/2025.05.13.653701; this version posted May 14, 2025. The copyright holder for this preprint (which was not certified by peer review) is the author/funder. This article is a US Government work. It is not subject to copyright under 17 USC 105 and is also made available for use under a CC0 license.

means, colored points demonstrate individual cell values for each run. N = 3 experiments for each graph; n = 15 fields of view for each condition per experiment for phagocytosis (E-H, L-M) or n = 80 cells for each condition per experiment for migration (A-C, I-K, N-O). Statistical analysis was assessed using the Kruskal–Wallis with Dunn multiple comparisons test correction: ns = not significant, *p < 0.05, **p < 0.01, ***p < 0.001, ****p < 0.0001. Error Bars represent SEM.

Supplementary Figure 3: Additional phagocytic uptake and cell motility data related to Figures 4 and 5. A, B, D, E) These graphs relate to the experiments presented in Figure 4. F-I) These graphs relate to the experiments presented in Figure 5. A, D, F, H) Average phagosome area per phagocytic cell (left) and average phagosome number per phagocytic cell (right) when treated with the respective drug (Blebbistatin, Cytochalasin D, Cilengitide) or when plated on PEG-PLL. B, E, G, I) Velocity (µm/min) (left) and maximum accumulated distance traveled (µm) (right) of cells when treated with the respective drug (Blebbistatin, Cytochalasin D, Cilengitide) or when plated on PEG-PLL. C) Persistence (left), velocity (µm/min) (middle), and maximum total accumulated distance traveled (µm) (right) readings for cells with and without blebbistatin treatment when no beads are present. For all graphs, black points demonstrate experiment means, colored points demonstrate individual cell values for each run. N = 3 experiments for each graph; n = 15 fields of view for each condition per experiment for phagosomes (A, D, F, H) or n = 50 cells for each condition per experiment for migration (B, E, G, I). Statistical analysis was assessed using the Kruskal–Wallis with Dunn multiple comparisons test correction: ns = not significant, *p < 0.05, **p < 0.01, ***p < 0.001, ****p < 0.0001. Error Bars represent SEM.

LIST OF ACCOMPANYING SUPPLEMENTARY MOVIES

Supplementary Movie 1: BV-2 cells migrating in media vs confinement for 8 hours. Scale bar = 50 microns.

Supplementary Movie 2: BV-2 cells migrating in media vs confinement ± CK-666 treatment for 8 hours. Scale bar = 50 microns.

bioRxiv preprint doi: https://doi.org/10.1101/2025.05.13.653701; this version posted May 14, 2025. The copyright holder for this preprint (which was not certified by peer review) is the author/funder. This article is a US Government work. It is not subject to copyright under 17 USC 105 and is also made available for use under a CC0 license.

Supplementary Movie 3: BV-2 cells migrating in media vs confinement ± Blebbistatin treatment for 8 hours. Scale bar = 50 microns.

Supplementary Movie 4: BV-2 cells migrating in media vs confinement ± Cytochalasin D treatment for 8 hours. Scale bar = 50 microns

Supplementary Movie 5: BV-2 cells migrating in media vs confinement ± Cilengitide treatment for 8 hours. Scale bar = 50 microns.

Supplementary Movie 6: BV-2 cells migrating in media vs confinement with FN or PEG-PLL coating for 8 hours. Scale bar = 50 microns.

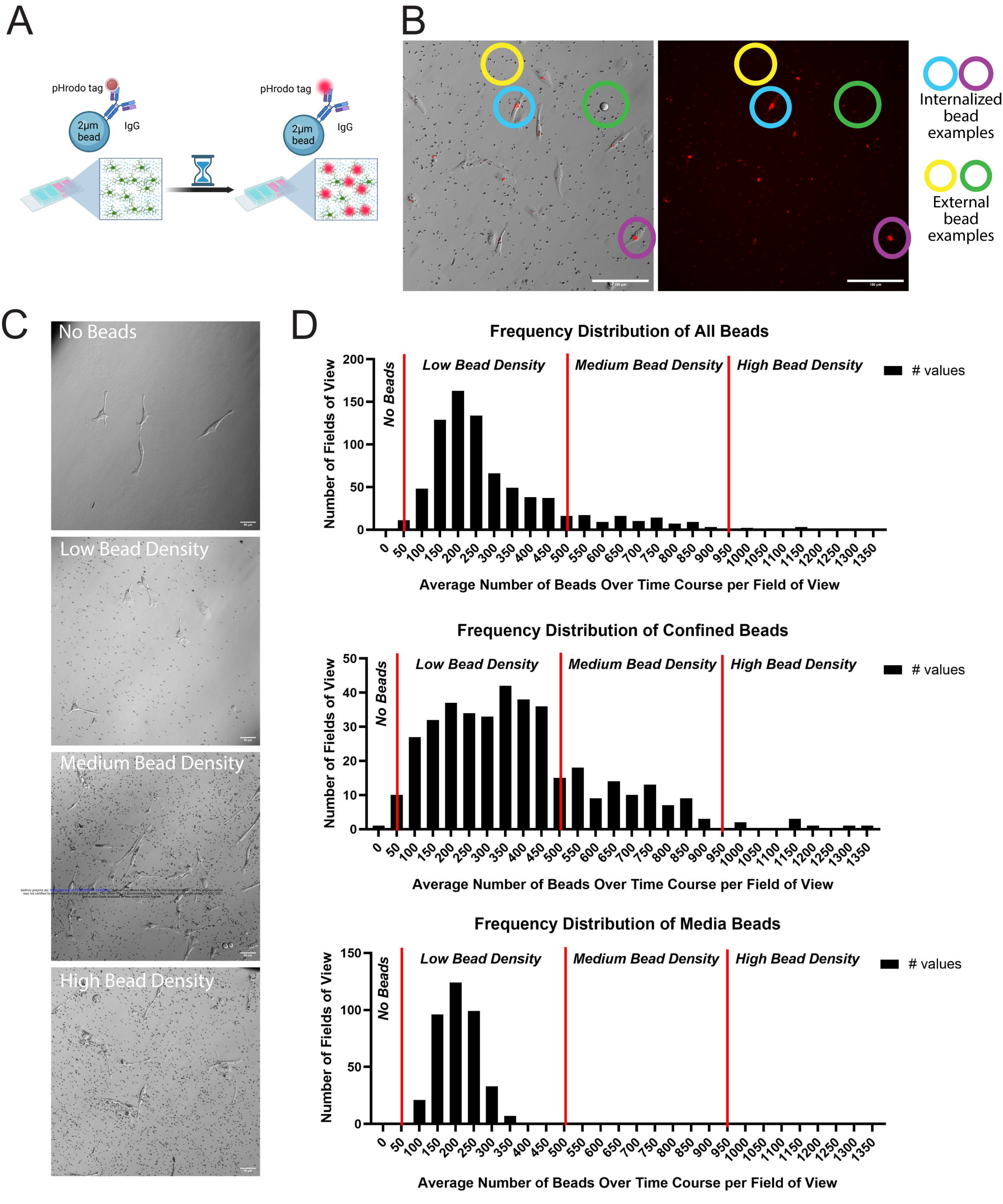

bioRxiv preprint doi: https://doi.org/10.1101/2025.05.13.653701; this version posted May 14, 2025. The copyright holder for this preprint (which was not certified by peer review) is the author/funder. This article is a US Government work. It is not subject to copyright under 17 USC 105 and is also made available for use under a CC0 license.

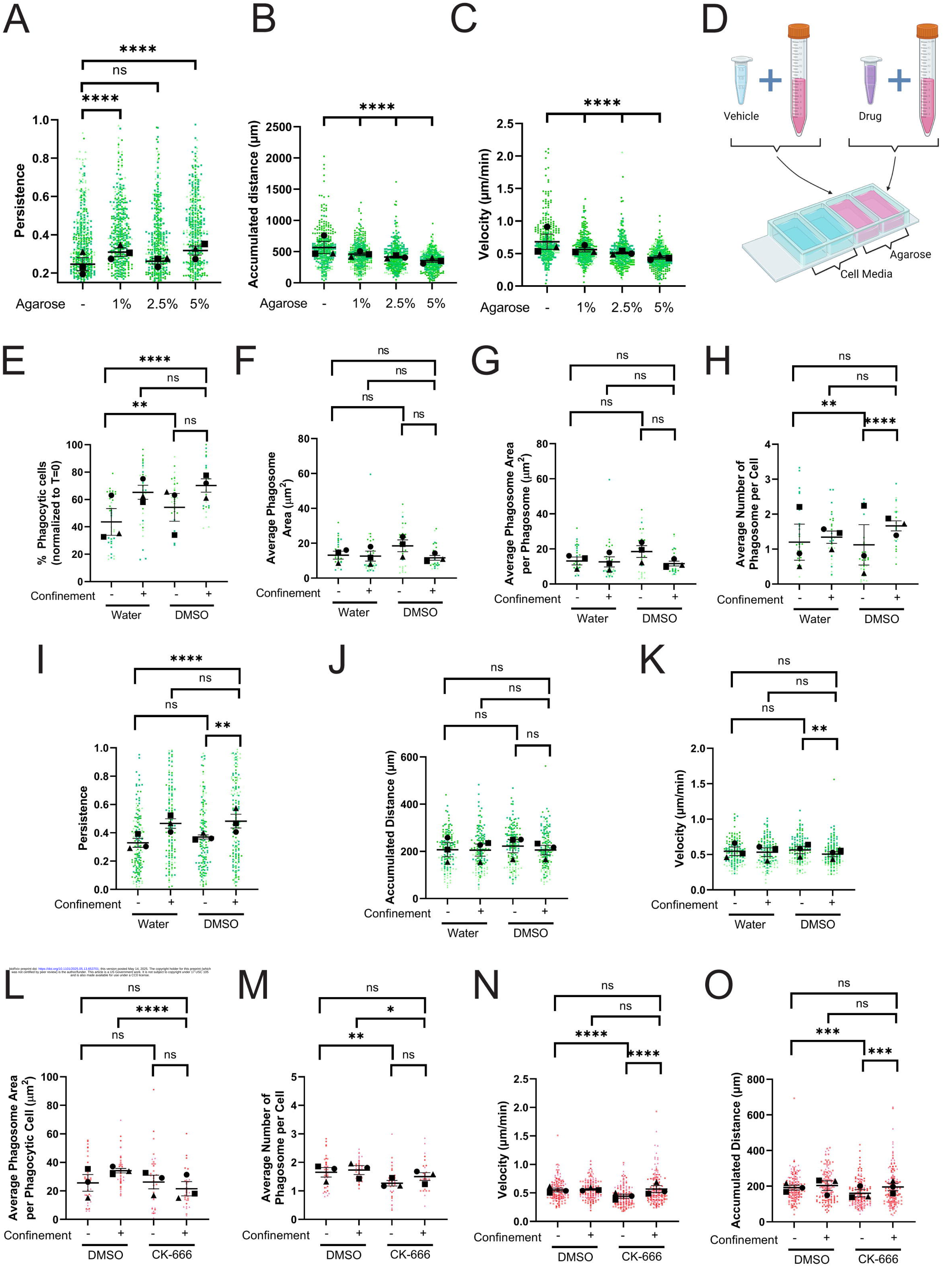

bioRxiv preprint doi: https://doi.org/10.1101/2025.05.13.653701; this version posted May 14, 2025. The copyright holder for this preprint (which was not certified by peer review) is the author/funder. This article is a US Government work. It is not subject to copyright under 17 USC 105 and is also made available for use under a CC0 license.

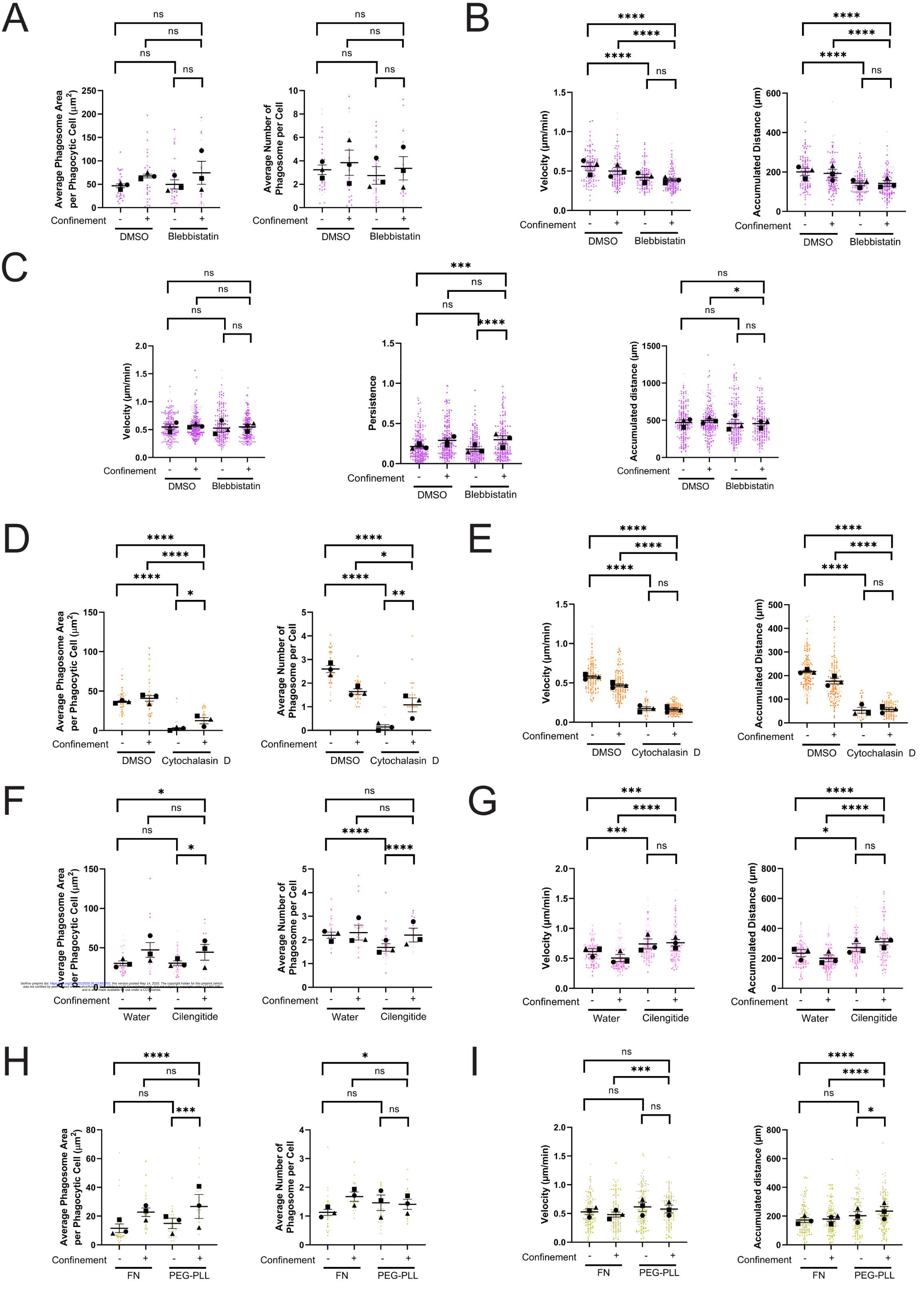

bioRxiv preprint doi: https://doi.org/10.1101/2025.05.13.653701; this version posted May 14, 2025. The copyright holder for this preprint (which was not certified by peer review) is the author/funder. This article is a US Government work. It is not subject to copyright under 17 USC 105 and is also made available for use under a CC0 license.
